# Draft Genome Sequence of Providencia rettgeri APW139_S1, an NDM-18-Producing Clinical Strain Originating from Hospital Effluent in South Africa

**DOI:** 10.1128/MRA.00259-19

**Published:** 2019-05-23

**Authors:** Noyise B. Ntshobeni, Mushal Allam, Arshad Ismail, Daniel G. Amoako, Sabiha Y. Essack, Hafizah Y. Chenia

**Affiliations:** a Discipline of Microbiology, College of Agriculture, Engineering and Science, University of KwaZulu-Natal, Durban, South Africa; b Sequencing Core Facility, National Institute for Communicable Diseases, National Health Laboratory Service, Johannesburg, South Africa; c Antimicrobial Research Unit, College of Health Sciences, University of KwaZulu-Natal, Durban, South Africa; University of Maryland School of Medicine

## Abstract

Providencia rettgeri is an opportunistic pathogen implicated in various clinical infections. Here, we report the genome sequence of a Providencia rettgeri strain isolated from hospital effluent in South Africa, which harbors the New Delhi metallo-β-lactamase (NDM) variant 18 gene (*bla*_NDM-18_). The 4,835,047-bp genome encodes a resistome and virulome that are of cardinal importance to Providencia infections.

## Abstract (precis)

Providencia rettgeri is an opportunistic pathogen implicated in various clinical infections. Here, we report the genome sequence of a Providencia rettgeri strain isolated from hospital effluent in South Africa, which harbors the New Delhi metallo-β-lactamase (NDM) variant 18 gene (*bla*_NDM-18_).

## ANNOUNCEMENT

Providencia rettgeri, an opportunistic pathogen belonging to the family Enterobacteriaceae, is implicated in various diseases (1). Notably, many recent unique isolates have been found to be carbapenemase producers carrying the New Delhi metallo-β-lactamase (NDM) gene (2). The NDM-18 variant is identical to NDM-1 except for a tandem repeat of 5 amino acids (QRFGD, amino acid positions 44 to 48 of NDM-1) (3, 4). Moreover, to date, there are only a few genome sequences of P. rettgeri available in public databases. Here, we report the first draft genome sequence of a *P. rettgeri* isolate encoding the *bla*_NDM-18_ gene.

*P. rettgeri* APW139_S1 was isolated in 2017 from hospital effluent originating from the pediatric ward of a rural hospital in the uMgungundlovu district of KwaZulu Natal Province, South Africa. The API 20E system (bioMérieux, France) was used to confirm culture identity of *P. rettgeri* APW139_S1. The APW139_S1 strain was grown on nutrient agar (Oxoid, England) and incubated overnight at 37°C prior to DNA extraction. Genomic DNA was extracted from 1 CFU of a visibly pure culture of the isolate using the GenElute bacterial genomic DNA kit (Sigma-Aldrich, St. Louis, MO, USA). A paired-end library (2 × 300 bp) was prepared using a Nextera XT DNA sample preparation kit, and whole-genome sequencing (WGS) was carried out on a MiSeq machine (Illumina, USA). The sequenced reads (1,476,722 reads) were quality trimmed and *de novo* assembled into 68 contigs (99× coverage and an *N*_50_ value of 215,431 bp) using Genomics Workbench version 10.1 (CLC bio, Qiagen, Aarhus, Denmark). Default parameters were used for all software unless otherwise specified. The NCBI Prokaryotic Genome Annotation Pipeline version 4.3 was used for annotation (5). The genome had a 4,835,047-bp genome size, a GC content of 40.50%, a total of 4,566 genes, a total of 4,487 coding sequences (CDS), 4,408 CDS coding genes, 79 RNA genes, 9 rRNAs, and 66 tRNAs.

CRISPRFinder (6) predicted two confirmed clustered regularly interspaced short palindromic repeat 1 (CRISPR1) arrays, both located on contig 2 (GenBank accession no. SHDG01000002).

The pathogenic bacterial database VFanalyzer (7) allowed identification of the critical virulence factors for adherence (*ecpA*, *ecpC*, *papD*, and *fimD*), invasion (*ibeC*, *cheB*, and *motA*), regulation (*phoQ*), toxin (*rtxB*, *rtxD*, and *rtxE*), efflux (*farB*), and endotoxin (*htrB* and *ipxK*), which contribute to the bacterium’s ability to adhere, invade the host, mediate adaptation to the host, secrete toxins, mediate resistance, and cause autoimmune-based host responses, respectively (8, 9).

The GoSeqIt tools Web platform (10) predicted an acquired antibiotic resistome, including genes coding for aminoglycoside [*aadA1* and *aac(6)-lb3*], fluoroquinolone/quinolone [*aac(6)-lb-cr*], macrolide [*mph(E)* and *msrE*], rifampin (*ARR-2*), sulfonamide (*sul1*), tetracycline [*tet(59)*], trimethoprim (*dfrA14*), and β-lactam (*bla*_OXA-2_, *bla*_OXA-10_, *bla*_NDM-18_, and *bla*_PER-7_) resistance. *In silico* WGS analysis indicated that the subclass B1 metallo-β-lactamase NDM-18 was plasmid encoded and contained on contig_68 (GenBank accession no. SHDG01000068). Comparative BLAST analysis of contig_68 indicated its similarity (query length, 100%; identity, ≥99.66%) to *bla*_NDM-1_-harboring plasmids of other Enterobacteriaceae species, such as Klebsiella pneumoniae plasmids pA1705 (GenBank accession no. MH909349), p362713 (accession no. MH909347), and p309074 (accession no. MH909346), all from China. To the best of our knowledge, this is the first report of the NDM-18 variant in *P. rettgeri*. The valuable information offered by genomics allows a better understanding of the resistance and pathogenicity of multidrug-resistant (MDR) *P. rettgeri* strains.

This study was approved by the Biomedical Research Ethics Committee (BE521/16), College of Health Sciences, University of KwaZulu-Natal.

### Data availability.

This whole-genome shotgun project has been deposited in DDBJ/ENA/GenBank under the accession no. SHDG00000000. The version described in this paper is the first version, SHDG01000000. The raw reads have been submitted to the SRA under accession no. SRR8591527.
